# Proteomic analysis of protein phosphatase Z1 from *Candida albicans*

**DOI:** 10.1371/journal.pone.0183176

**Published:** 2017-08-24

**Authors:** Bernadett Márkus, Krisztina Szabó, Walter P. Pfliegler, Katalin Petrényi, Enikő Boros, István Pócsi, József Tőzsér, Éva Csősz, Viktor Dombrádi

**Affiliations:** 1 Proteomics Core Facility, Department of Biochemistry and Molecular Biology, Faculty of Medicine, University of Debrecen, Debrecen, Hungary; 2 Department of Medical Chemistry, Faculty of Medicine, University of Debrecen, Debrecen, Hungary; 3 Department of Biotechnology and Microbiology, Faculty of Science and Technology, University of Debrecen, Debrecen, Hungary; Centre National de la Recherche Scientifique, FRANCE

## Abstract

Protein phosphatase Z is a “novel type” fungus specific serine/threonine protein phosphatase. Previously our research group identified the *CaPPZ1* gene in the opportunistic pathogen *Candida albicans* and reported that the gene deletion had several important physiological consequences. In order to reveal the protein targets and the associated mechanisms behind the functions of the phosphatase a proteomic method was adopted for the comparison of the *cappz1* deletion mutant and the genetically matching QMY23 control strain. Proteins extracted from the control and deletion mutant strains were separated by two-dimensional gel electrophoresis and the protein spots were stained with RuBPS and Pro-Q Diamond in order to visualize the total proteome and the phosphoproteome, respectively. The alterations in spot intensities were determined by densitometry and were analysed with the Delta2D (Decodon) software. Spots showing significantly different intensities between the mutant and control strains were excised from the gels and were digested with trypsin. The resulting peptides were identified by LC-MS/MS mass spectrometry. As many as 15 protein spots were found that exhibited significant changes in their intensity upon the deletion of the phosphatase and 20 phosphoproteins were identified in which the level of phosphorylation was modified significantly in the mutant. In agreement with previous findings we found that the affected proteins function in protein synthesis, oxidative stress response, regulation of morphology and metabolism. Among these proteins we identified two potential CaPpz1 substrates (Eft2 and Rpp0) that may regulate the elongation step of translation. RT-qPCR experiments revealed that the expression of the genes coding for the affected proteins was not altered significantly. Thus, the absence of CaPpz1 exerted its effects *via* protein synthesis/degradation and phosphorylation/dephosphorylation. In addition, our proteomics data strongly suggested a role for CaPpz1 in biofilm formation, was confirmed experimentally. Thus our unbiased proteomic approach lead to the discovery of a novel function for this phosphatase in *C*. *albicans*.

## Introduction

Protein phosphatase Z (PPZ) is a novel member of the Ser/Thr specific phosphoprotein phosphatase family that was identified by molecular cloning [1]. This phosphatase has a distinctive phylogenetic distribution as it is specific for fungi. It was discovered and has been extensively characterized in the model organism *Saccharomyces cerevisiae* that possesses two paralogs, Ppz1 and Ppz2 phosphatase [2]. PPZ orthologs have been also described in *Schizosaccharomyces pombe* [3], *Neurospora crassa* [4], *Debaryomyces hansenii* [5], *Aspergillus nidulans* [6] and the opportunistic pathogen *Candida albicans* [7].

All of the known PPZ enzymes consists of a well conserved C-terminal catalytic domain, and a more variable intrinsically disordered N-terminal regulatory domain. Based on its amino acid sequence and the recently determined three dimensional structure [8] the catalytic domain of *C*. *albicans* CaPpz1 is very similar to the catalytic subunit of protein phosphatase 1 (PP1c), suggesting that the PPZ subfamily evolved from PP1c *via* the fusion with a flexible N-terminal segment. However, due to several unique structural features—including a specific C-terminal helix—a number of point mutations in important binding sites, and the presence of its N-terminal tail [8] CaPpz1 exhibits distinct regulation and has different physiological functions in comparison to the classical PP1c termed Glc7 in *C*. *albicans*.

The analysis of the *cappz1* deletion mutant revealed that the *Candida* phosphatase, like its budding yeast counterpart, was involved in the regulation of cation homeostasis, cell wall biosynthesis, and membrane potential [9], as well as in the oxidative stress response [6]. In addition to the yeast-specific functions the absence of the CaPpz1 enzyme affected the adherence, morphogenesis [10] and the virulence of the pathogenic fungus [9,11]. Thus in the past few years the pertinent functions of CaPpz1 were identified, and its regulation by the CaCab3 protein was also revealed [12]. However, practically nothing is known about the mechanisms of its action.

Based on analogies with *S*. *cerevisiae* and other tested fungal species we may pinpoint some of the potential PPZ targets. In an earlier study the translational elongation factor 1Bα (eEF1Bα) was identified as the direct substrate of Ppz1,2 after *in vivo* phosphorylation of budding yeast cells, and classical Edman protein sequencing of a selected protein spot [13]. Furthermore, a number of protein substrates have been utilized for the *in vitro* assay of PPZ activity that include: myelin basic protein, histone 2A [14], Reg1 [15], and myosin light chain 20 [12], although the physiological relevance of these substrate proteins is questionable.

The detailed analysis of budding yeast deletion mutants may also reveal some of the physiological targets of PPZ phosphatases. For example the tolerance of the *ppz1* and *ppz1*,*2* budding yeast mutants to Na^+^ and Li^+^ ions was explained in part by the elevated expression of the *ENA1* gene that codes for the major Na^+^-transporter, and the enhancement of the Ena1 mediated cation efflux rate [16]. On the other hand, in the salt tolerant *D*. *hansenii* the deletion of the *DhPPZ1* increased the expression of the *DhEHA1* Na^+^/H^+^ antiporter gene [5]. In addition, an *ENA1*-independent component of cation tolerance in the *ppz1*,*2* mutant *S*. *cerevisiae* was attributed to the activation of Trk1 and Trk2 K^+^ transporters [17]. The tolerance of the phosphatase deficient strain to the toxic cations, like spermine and hygromycin B was explained by the reduced membrane potential that was related to the altered K^+^ transport [17]. Finally, the sensitivity of the mutant to the cell wall biosynthesis blocking agents like caffeine and calcofluor white was associated with the elevated turgor pressure and osmotic instability caused by the increased intracellular K^+^ concentration [17]. According to this unifying model the absence of the *PPZ1*,*2* gene products facilitates K^+^ influx; that changes membrane potential, results in osmotic instability, and induces compensatory Na^+^ and H^+^ efflux; the latter is responsible for alkalinization inside the cells and a subsequent change in the pH-dependent gene expression. A genetic interaction between *S*. *cerevisiae PPZ1*,*2* phosphatases and the protein kinase C regulated *SLT2/MPK1* mitogen-activated protein kinase [18] that is regulating the cell wall integrity (CWI) pathway [19], explains the negative correlation between the cell wall integrity and osmotic stability phenotypes of the *ppz1* and *slt2* mutants [20].

Previously we have reported that the *C*. *albicans* CaPpz1 was able to complement the missing *S*. *cerevisiae* Ppz1 phosphatase in the *ppz1* mutant and mimicked the effect of Ppz1 in a MAP kinase deficient *slt2* deletion strain [9]. It was also found that the PPZ phosphatase orthologs in *A*. *nidulans*, *C*. *albicans*, and *S*. *cerevisiae* [6] as well as in *Aspergillus fumigatus* [21] were involved in the oxidative stress response.

Thus, from the available literature one can suggest a score of possible CaPpz1 targets in *C*. *albicans* that may explain the observed physiological functions of the phosphatase. However, it has to be kept in mind that these analogy-based assumptions have to be proven by solid experimental evidence. For the identification of the protein targets for CaPpz1 we initiated an unbiased proteomic approach that reveals the proteins whose phosphorylation or expression level is affected by the deletion of the *CaPPZ1* gene.

## Materials and methods

### Fungal strains and growth conditions

We compared two genetically matching *C*. *albicans* strains in the present study. The control strain QMY23 [22] was kindly provided by Alexander Johnson, and has the following genotype: *his1Δ/his1Δ*, *leu2Δ*::*C*. *dubliniensis HIS1/leu2Δ*::*C*. *maltosa LEU2*, *URA3/ura3Δ*::*imm434*, *IRO1/iro1Δ*::*imm434*. The *cappz1* deletion mutant with the genotype: *ura3Δ-iro1Δ*::*imm434/URA3-IRO1*, *his1Δ/his1Δ*, *leu2Δ/leu2Δ*, *ppz1Δ*::*C*. *dubliniensis HIS1/ppz1Δ*::*C*. *maltosa LEU2* was generated in our laboratory [9]. Thus, in the KO strain both alleles of the *CaPPZ1* gene are deleted, otherwise its genetic background would have been identical to the control strain. Pre-cultures of the two strains were grown overnight from single colonies in YPD medium (10 g yeast extract, 20 g peptone, and 20 g glucose in 1 L solution) at 37°C without shaking. The main cultures were diluted to OD_640_ 0.1 with YPD and were grown under identical conditions with shaking (140 rpm) until the OD_640_ of the *cappz1* strain reached 0.8. Then the cells were collected by centrifugation at 3273 x g, 4°C for 10 min. A typical growth curve is presented in the S1 Fig. The pellets were resuspended and washed twice with 1 ml cold phosphate-buffered saline (PBS). The washed cells were collected by centrifugation at 7750 x g, 4°C for 10 min, were quick-frozen in liquid nitrogen, and were stored at -70°C until use. The deletion of the *CaPPZ1* gene has been confirmed in all of the samples by PCR performed according to Ádám *et al*. [9].

### Proteomics and phosphoproteomics

#### Protein sample preparation and purification

Three independent samples originating from control and mutant cells, respectively, were resuspended in 1 ml lysis buffer containing 50 mM NaH_2_PO_4_ (pH = 7.4) 1 mM EDTA, 5% glycerol, complete EDTA-free protease inhibitor mixture (Roche), 5 mM benzamidine, 10 nM leupeptine, 1 mM PMSF, and 1 mM MnCl_2_. To the cell suspension 500 μl glass beads (1 mm ∅, Marienfeld) were added, and were vortexed at the maximal speed 3 times for 1 min, with 2 min intervals cooling in ice. Cell debris were collected by centrifugation (500 x g, 10 min, 4°C), and the protein concentration of the supernatants was determined with the method of Bradford [23]. Samples containing 450 μg protein underwent a cleanup by Ready-Prep 2-D CleanUp Kit (Bio-Rad) according to the manufacturer’s protocol. After precipitation and centrifugation, each pellet was dried and resuspended in 450 μl rehydration buffer (7 M urea, 2 M thiourea, 4 m/v% CHAPS, 1% DTT, 2 v/v% Bio-Lyte and 0.001% bromophenol blue) and used immediately for two-dimensional electrophoresis. In order to have preparative gels for spot picking a mix group was also created by the combination of equal amounts of control and mutant protein samples and was processed as described before.

#### Two-dimensional (2D) gel electrophoresis

24 cm, pH 4–7 IPG strips with immobilized pH gradient (Bio-Rad) were passively rehydrated with 450 μl sample at 20°C overnight. The isoelectric focusing (IEF) was performed by applying 300 V for 3 hours, gradually increased to 3500 V in 5 hours and then held at 3500 V for 18 hours. After IEF the IPG strips were equilibrated in equilibration buffer (500 mM Tris-HCl, pH = 8.5, 6 M urea, 2% SDS, 20% glycerol) containing 0.6% dithiothreitol (DTT) for 15 min, and then in equilibration buffer containing 1.2% iodoacetamide (IAA) for 15 min. In the second dimension the strips were laid on the top of 12% SDS-polyacrylamide gels and covered with agarose (Bio-Rad). No molecular weight marker was applied. In a Protean Plus Dodeca Cell (Bio-Rad) the electrophoresis was carried out simultaneously for each gel at 100 mA per gel for 24 hours until the bromophenol blue dye reached the bottom of the gel. Proteins were first stained with Pro-Q Diamond dye (Thermo Life Technologies) according to manufacturer’s instructions, washed with water and scanned with Pharos FX Plus Molecular Imager (Bio-Rad) in order to visualize the phosphoproteome. For excitation 532 nm wavelength light was used and the image was recorded at 615 nm; the scanning was done at 100 micrometres resolution. The gels were stained again with in-house prepared RuBPS fluorescent protein staining dye solution in 20% ethanol overnight [24] to reveal the total proteome, than were washed in post-fix solution containing 7% acetic acid and 10% methanol. Gel images were recorded as before. The three biological replicates of the control and mutant samples as well as the mix groups were processed together on the same day.

#### Quantitative analysis of 2D gels

Protein spot patterns were detected using Delta2D (Decodon) software version 4.4. The gel images were grouped as follows: the control (Ctrl), the *cappz1* deletion mutant (Mut) and finally, the mix group (Mix). Three projects were created: (i) in the first project the Pro-Q Diamond-stained Ctrl and Mut gel images were studied, (ii) in the second project the RuBPS-stained Ctrl and Mut gel images were examined, while (iii) in the third project all spots were transferred to Mix gel images for spot excision. Protein spots from all of the Ctrl and Mut gels were matched using the exact mode matching protocol and the group warping strategy of Delta2D software. The union mode was used to generate the fused images containing each spot present on all of the gels included into each project. All spots were quantified and the total quantity in all of the spots was taken as 100%. The spot intensity was normalized according to the total intensity of each spot in all gels and was given as normalized spot volume (%v) compared to the total intensity. The fold change of mean normalized spot volume of three Mut and three Ctrl gels (Mut/Ctrl) was calculated and the significance of differences was assessed automatically by the Delta2D software using Student’s t-test. The Quantitation table generated automatically by the software was exported to Microsoft Excel 2010 (Microsoft Inc.). All the relevant raw data are publicly accessible at the following link under https://figshare.com/account/home#/projects/23773.

#### In-gel digestion of proteins

Those protein spots that exhibited significantly different (p < 0.05) normalized spot volumes between the Ctrl and Mut groups using either Pro-Q Diamond or RuBPS staining were cut out manually from the mix gel with a pipette tip and were subjected to in-gel digestion. Initially, the gel spots were destained using a 1:1 ratio of 25 mM ammonium bicarbonate pH = 8.5 and 50% acetonitrile, than were reduced by 20 mM DTT for 1 hour at 56°C. Next alkylation was accomplished by 55 mM IAA for 45 min at room temperature in the dark, then an overnight digestion was performed with 100 ng stabilized MS grade trypsin (ABSciex) at 37°C. The reaction was stopped by concentrated formic acid (FA). The tryptic peptides diffused out of the gel pieces were dried in speed-vac concentrator (Thermo Scientific).

#### Protein identification by mass spectrometry

For protein identification the peptides were re-dissolved in 10 μl 1% FA and were separated on Easy nLCII (Bruker) nanoHPLC using a 90 min water/acetonitrile gradient at 300 nl/min flow rate. The peptide mixture was loaded onto a Zorbax 300SB-C18 desalting column (5 mm x 0.3 mm, 5 μm particle size, Agilent). Thereafter, the peptides were separated on a Zorbax 300SB-C18 analytical column (150 mm x 75 μm, 3.5 μm particle size, 300 Å pore size, Agilent). The mobile phase A: 0.1% FA in LC-MS grade water (Sigma), was mixed with the mobile phase B: acetonitrile (Sigma) containing 0.1% FA. During the separation the percentage of phase B was increased from 0% to 100% in 60 min, then held at 100% for 10 min, decreased to 0% in 2 min, and finally was held at 0% for 18 min.

The peptides eluted from the analytical column were analyzed in a 4000 QTRAP (ABSciex) mass spectrometer recording positive ion mode MS/MS spectra. The Information Dependent Acquisition (IDA) method was selected. After the first mass scan (mass range 400–1700 amu), an enhanced resolution separation was carried out to establish the charge state of the two most intensive precursor ions. For protein identification collision-induced dissociation (CID) spectra were obtained in enhanced product-ion mode (mass range 100–1900 amu) at scan rate of 4000 amu/s and the rolling collision energy was applied with the maximum of 85 eV. The cycle time was 5.4 sec. The spray voltage was 2800 V, ion source gas was 50 psi, the curtain gas was 20 psi and the source temperature was 70°C.

Based on the acquired MS/MS spectra proteins were identified with the ProteinPilot 4.5 software (ABSciex) that used the UniProt/Swiss-Prot database (2015. 07. version, 548872 sequence entries) and the Biological modification table implemented in the software, containing more than 70 frequent biological protein modifications. A minimum of two peptides identified with at least 95% confidence, where at least four b or y ions in ion series were present, were used for protein identification. In those cases where the protein identification was not successful with ProteinPilot, a MASCOT search was carried out using the NCBInr database. The type of cleavage enzyme was determined as trypsin. The missed cleavage in both cases was set to maximum 1. The following variable modifications were defined: oxidation of methionine (Met) and carbamidomethylation of cysteine (Cys). In those cases where the identified protein was not assigned to the *C*. *albicans* strain used in our analyses, or was identified as hypothetical protein, a blastp (https://blast.ncbi.nlm.nih.gov/Blast.cgi?PAGE=Proteins) search was performed on the NCBI non-redundant protein database to identify the same peptide sequences in the *C*. *albicans* SC5314/ATCC MYA-2876 strain. All of our sequence annotations were confirmed by an independent data-mining of the CGD database (http://www.candidagenome.org/; *C*. *albicans* SC5314 Assembly 22 version s06-m01-r01, posted on February 2, 2016) in two steps. First a blastp search was carried out at customs settings, than the 13 short peptide sequences that were missed in the first screen were one by one manually located in the individual protein sequences.

### Gene expression studies

#### RNA isolation

Cells (QMY23, *cappz1*) were lyophilized for 4 hours. The lyophilized samples were first milled by sterile toothpick, then were suspended in 1 ml TRIzol reagent (Invitrogen) and finally vortexed for 3 minutes at 25°C. Cell debris were removed by centrifugation (11750 x g, 10 min, 4°C) and the lysates were extracted by the addition 0.2 volume of chloroform (VWR) for 10 minutes. After centrifugation (11750 x g, 10 min, 4°C) the upper aqueous phase was transferred into a new tube and isopropanol (VWR) was added in 1:1 ratio. After 10 min incubation at 25°C, the precipitated RNA was collected by centrifugation (11750 x g, 10 min, 4°C) and the pellet was washed by cold 70% ethanol. After air-drying the pellets in sterile box, samples were dissolved in nuclease free water (VWR) for 10 min at 55°C. RNA concentration and purity were checked by NanoDrop (Thermo Scientific) and agarose gel electrophoresis.

#### RT-qPCR

3 μg RNA were treated with RNase free DNase I (Fermentas) according to the protocol of the manufacturer. For cDNA synthesis 2 μg DNase-treated RNA was incubated with 1 μl Oligo (dT) Primer (Promega) for 5 min at 70°C in 11 μl final volume. Then 5 μl 5 x M-MLV Reaction buffer, 5 μl 2 mM dNTP, 1 μl Recombinant RNasin ® Ribonuclease Inhibitor (40 U/μl) (Promega), 1.5 μl M-MLV Reverse Transcriptase (200 U/μl) (Promega) and nuclease free water were added and samples were incubated for 1 hour at 37°C. cDNA samples were stored at -20°C until use. For each cDNA to be tested specific primer pairs were designed with the Primer3 version 0.4.0 (http://bioinfo.ut.ee/primer3-0.4.0/primer3/) and Oligo Analyser version 1.0.2 softwares, and were purchased from Sigma. Each primer pair was tested by PCR (S2 Fig). The oligonucleotide sequences and optimal PCR conditions are summarised in S1 Table. For quantitative real time PCR primers were diluted in nuclease free water to 10 μM. For one reaction 5 μl Master Mix (2x qPCRBIO SyGreen Mix Lo-ROX, Nucleotest BIO), 1 μl primer mix (forward and reverse primers), and 2 μl nuclease free water was mixed. 8 μl of the reaction mix was added to 2 μl 10 ng/μl cDNA in one well of the 4titude Framestar® 480/384 plate that was covered with qPCR Adhesive Seal (Nucleotest BIO). Three parallel measurements were made with every sample in the LightCycler®480 II instrument (Roche). The template program consisted of the following steps: pre-incubation (1 cycle), amplifications (40 cycles), melting curve (1 cycle) and cooling. The acquisitions were achieved at 60°C and the target temperature was 95°C (Ramp Rate: 4.8°C/s). Real time detection was achieved by SYBR Green intercalation, and the T_m_ (melting temperature) as well as Cp (cycle number at detection threshold /crossing point) data were determined by the built in LCS480 1.5.0.39 Software. The data were normalized to Actin (*ACT1* gene) expression. SD, p values and fold change (*cappz1*/QMY23 Control) values were calculated and the statistical analysis was performed in Microsoft Excel 2010 (Microsoft Inc).

### Investigation of biofilm formation

#### Biofilm formation assay

*In vitro* biofilm formation was measured in Spider medium (1% mannitol, 1% nutrient broth, 0.2% K_2_HPO_4_, pH = 7.2) and bicarbonate-free RPMI-1640 (Sigma-Aldrich) by growing the biofilm on the bottom of untreated 12-well polystyrene plates (Multiwell Cell Culture Plates, VWR), as follows. *C*. *albicans* strains were grown for 18 hours in Sabouraud Dextrose Broth (SDB) at 37°C and wells were inoculated with 2 x 10^6^ yeast cells in 2 ml Spider or RPMI medium. Incubation of the plates was carried out at 37°C for 24 hours with 50 rpm agitation. A subsequent washing step after initial adhesion was not applied, as the two strains were shown to have different adhesion characteristics [10], consequently washing would have affected the results of the biofilm assay. After incubation, planctonic and easily detaching cells as well as the medium were carefully removed, and biofilms were washed twice with 1 ml PBS. Planktonic cells were collected in microcentrifuge tubes. The dry biofilm mass and the mass of planktonic/detached cells were measured according to the method of Nobile *et al*. [25]. Biofilm assays were carried out in triplicates from three independent inocula and each strain/medium combination was tested on an individual plate in all 12 wells. Thus, for each combination, biofilm dry biomass to total dry biomass ratios were calculated for 36 individual wells.

#### Crystal violet staining

As an independent way to measure biofilm growth Crystal violet (CV) staining was used. Strains were grown under the conditions described above, for 24 hours with 50 rpm agitation, in 24-well polystyrene plates (Linbro Tissue Culture Multiwell Plates, ICN Biomedicals). CV staining assays were carried out in triplicates from three different inocula and each strain/medium combination was tested in 12 wells of the tissue culture plates. Staining, washing, distaining and absorbance measurements were conducted as described by Jin *et al*. [26].

#### Microscopy

Samples of biofilms formed in 12-well plates were temporary mounted on microscope slides and photographed using an Olympus BD40 microscope equipped with an Olympus 40X lens and a digital microscope camera, with the Olympus DP Controller software, with phase contrast illumination.

#### Statistical analysis

To compare the values obtained for biofilm dry biomass, biofilm to total biomass ratio, and absorbance values after CV staining, Student’s 2-sample t-test or Welch’s test (depending on the equality of variance) was applied. A p value < 0.05 was considered significant.

### Bioinformatics

Potential protein phosphorylation sites were identified in *C*. *albicans* proteins by Motif scan (http://scansite.mit.edu/motifscan_seq.phtml) screening at high and medium stringency settings [27]. Interactions between the *S*. *cerevisiae* orthologs of the *C*. *albicans* proteins were analyzed by the String version 10.5 (https://string-db.org) with default settings at medium stringency [28].

## Results and discussion

### Examination of CaPpz1 by proteomic methods

Two dimensional (2D) electrophoresis is an unbiased method for the examination of proteins present in adequate amount in biological samples [29]. After separation differential staining procedures facilitate the detection of native and post-translationally modified proteins in the same 2D gel [30]. In this study we investigated the functional consequences of the deletion of the *CaPPZ1* gene in *C*. *albicans* by using the combination of phosphoprotein and general protein staining of control QMY23 (Ctrl) and deletion mutant *cappz1* (Mut) strains.

#### Effects of CaPPZ1 deletion on protein phosphorylation

**Fig 1:**
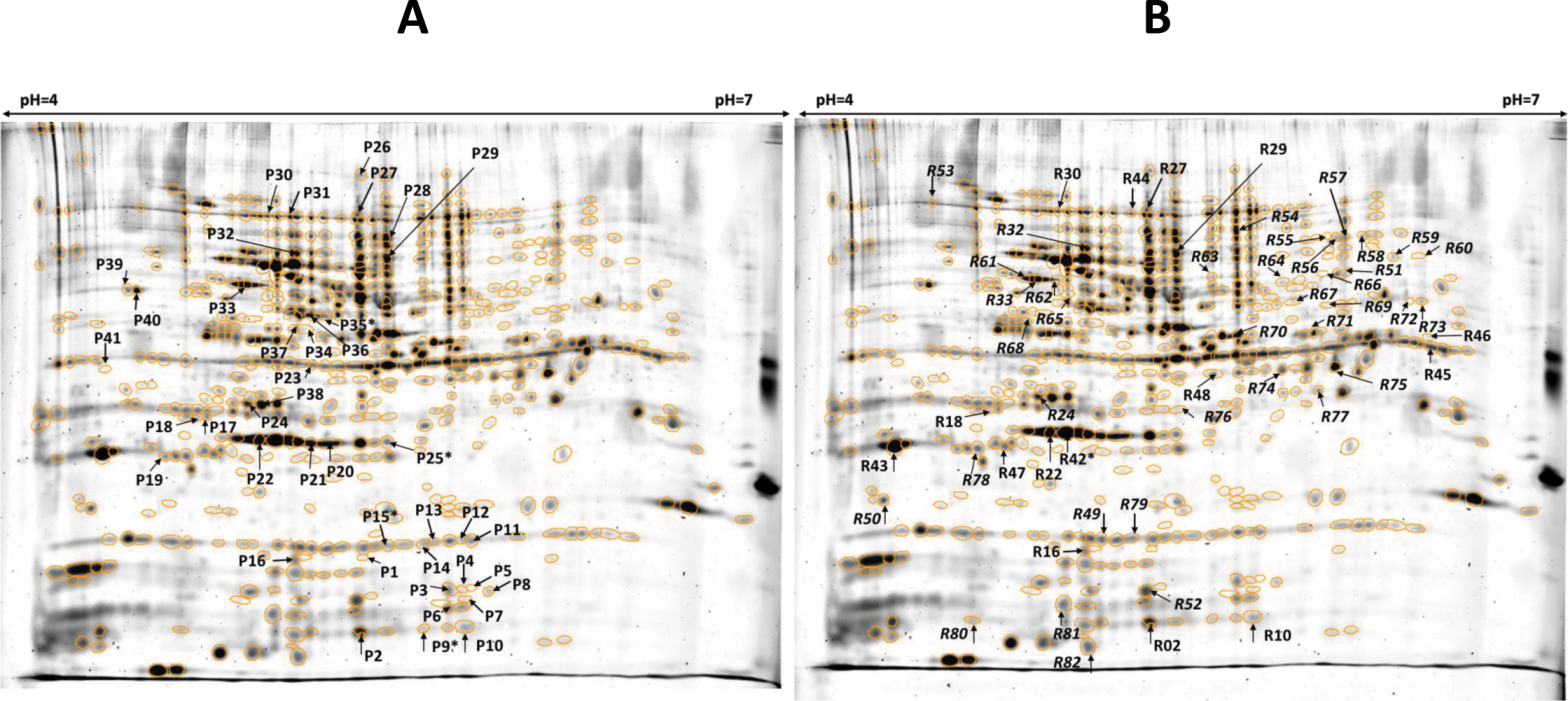
**Fused images of three Pro-Q Diamond (a) and three RuBPS (b) stained gels**. The arrows point to spots with significantly altered normalized spot volumes in the *cappz1* mutant *C*. *albicans* strain. Letters P or R in front of spot IDs indicate the staining methods. In the spots labelled with * more than one protein was found.

Phosphoproteins were visualized by the Pro-Q Diamond staining of 2D gels. By the combination of three independent Ctrl as well as Mut gel images a fused image was generated that contains all of the 473 Pro-Q Diamond stained spots present in Ctrl and Mut gels (Fig 1A). Then the mean normalized spot volume for each corresponding spots of Ctrl and Mut gels was determined and all of the 41 spots which exhibited significant (p < 0.05) difference between the two samples were excised from the Mix gel and were subjected to LC-MS/MS-based protein identification. The peptide sequences found in 27 separate spots could be determined (S2 Table). In most of these spots a single *C*. *albicans* protein was detected but four spots (9, 15, 25 and 35) contained two or more polypeptides. The latter were excluded from further analyses as it was impossible to judge that the phosphorylation of which protein was responsible for the observed intensity changes. The unambiguously identified proteins are listed in S3 Table in an alphabetical order. Our data revealed that four of the phosphorylated proteins were detected in two separate spots. Identical gene products (Rpp0, Lia1-like and Uba1) were found in three pairs of the spots, while the peptidyl-prolyl cis-trans isomerase was represented by two distinct isoenzymes (Cpr3 and Cyp5 in spots 2 and 7, respectively). Thus, in 23 spots we identified 20 individual proteins whose phosphorylation was significantly altered in the absence of the CaPpz1 phosphatase. It should be noted that in five of these proteins (Rpp0, Dug1-like, Eft2, Rad23 and Tkl1) a clearcut increase of phosphorylation was observed, as it can be expected when a phosphatase is knocked out. The case of ubiquitin-activating enzyme E1 (Uba1) was more complicated since in the Mut sample the phosphoprotein specific staining was more intensive in one spot (30) and was diminished in another (31). It is possible that these spots represent hyperphosphorylated and hypophosphorylated forms of the enzyme, respectively, in agreement with a pI shift between the two spots (Fig 1A). In the additional 14 proteins tested a reduced Pro-Q Diamond staining was recorded that may reflect some indirect effects of CaPpz1. However, one can not exclude the possibility that changes in the protein levels may mask or distort the changes caused by phosphorylation.

#### Effects of CaPPZ1 deletion on protein levels

In order to examine the changes in the protein levels upon *CaPPZ1* deletion the Pro-Q Diamond stained Ctrl and Mut gels were washed and then stained with RuBPS, a dye that labels the proteins regardless of the post-translational modifications. For the evaluation of the results the same strategy was applied as described in the previous section. The fused image generated from three replicates of Ctrl and Mut RuBPS-stained gels contained 519 spots, from which 52 exhibited statistically significant changes in normalized spot volumes between the two experimental groups (Fig 1B). 20 spots with more than 1.5 fold change were excised from the Mix gel and were analyzed by mass spectrometry. In 15 of the spots the protein identification was successful; 13 contained a single protein, while the elongation factor Cef3 was found in spots 27 and 44 (S2 Table). Thus, in 15 spots we identified 14 individual proteins that have a more than 1.5 fold change of their cellular level after the elimination of the *CaPPZ1* gene (S4 Table).

#### Evaluation of the proteomic results

The results of Pro-Q Diamond and RuBPS staining were combined and summarised in Table 1. Based on the changes in the protein content and protein phosphorylation the proteins were divided into four main categories. In Group 1 there are 5 proteins that have no phosphorylated counterparts. In this group the amount of Bmh1 and Efb1 decreased, while the amount of Ade12, Idh1 and Ure2 proteins increased in the knock out strain.

**Table 1:** Summary of changes in protein levels, protein phosphorylation and gene expression in *Candida albicans* in response to *CaPPZ1* gene deletion.

Protein^1^^,^^2^	Spot ID	RuBPS staining		Pro-Q Diamond staining		Gene expression	
		Ratio	p value	Ratio	p value	Ratio	p value
***Group 1***							
Ade12	46	**2.60**	**0.04**	n.a.	-	1.09	0.82
Bmh1	47	**-1.97**	**0.01**	n.a.	-	-1.07	0.44
Efb1	43	**-2.37**	**0.002**	n.a.	-	1.38	0.33
Idh1	45	**2.76**	**0.02**	n.a.	-	-1.02	0.95
Ure2	48	**1.95**	**0.0004**	n.a.	-	-1.28	0.36
***Group 2***							
Cef3*	44	**-7.08**	**0.004**	n.a.	-	**1.43**	**0.01**
	27	**-3.63**	**0.02**	**-2.38**	**0.008**		
Cpr3	02	**-2.20**	**0.01**	**-2.59**	**0.018**	1.05	0.36
Hsp70/Ssa1	32	**-1.52**	**0.04**	**-3.32**	**0.024**	1.17	0.59
Rpl20B	10	**-2.21**	**0.02**	**-4.81**	**0.019**	1.50	0.10
Tkl1	29	**1.84**	**0.01**	**1.78**	**0.045**	-1.19	0.44
Tsa1	16	**-2.23**	**0.002**	**-2.18**	**0.028**	1.14	0.23
Yst1	22	**-2.35**	**0.04**	**-2.79**	**0.041**	1.79	0.06
***Group 3***							
Aip2	34	-1.25	0.51	**-2.05**	**0.026**	1.67	0.18
Cyp5	07	-2.62	0.09	**-2.02**	**0.0003**	1.61	0.15
Dug1-like	36	-1.25	0.51	**1.37**	**0.008**	1.13	0.29
Eft2	28	-1.11	0.69	**2.79**	**0.008**	1.10	0.63
Guk1	03	-1.84	0.13	**-4.57**	**0.003**	1.87	0.05
Ham1	01	1.34	0.46	**-2.79**	**0.034**	1.17	0.49
Rad23	39	-1.24	0.37	**1.95**	**0.006**	-1.26	0.17
Rpl9B	13	-1.69	0.19	**-3.23**	**0.049**	1.32	0.10
Rpp0*	20	1.74	0.15	**2.56**	**0.046**	1.20	0.67
	21	-1.08	0.82	**2.63**	**0.005**		
Rps7A	04	-4.98	0.12	**-2.58**	**0.047**	**1.22**	**0.03**
Tif1	23	1.09	0.77	**-2.05**	**0.014**	1.44	0.16
***Group 4***							
Lia1-like*	17	-1.06	0.75	**-3.09**	**0.019**	1.54	0.10
	18	**-1.59**	**0.02**	**-2.32**	**0.008**		
Uba1*	30	**1.59**	**0.03**	**1.90**	**0.006**	1.03	0.98
	31	1.21	0.56	**-2.31**	**0.037**		

* The protein was identified in two separate spots of 2D gels. n.a. not applicable, as no phosphoprotein specific staining can be detected. Significant changes (p < 0.05) are shown in bold face.

Based on

^1^http://www.uniprot.org/ and

^2^ http://www.candidagenome.org/.

The members of a Group 2 have been characterized as phosphoproteins (Table 1). In these 7 proteins the Pro-Q Diamond and RuBPS staining of the coinciding spots changed in the same way, indicating that only the amount of the phosphoprotein was affected without changing the level of phosphorylation. The phosphatase deletion diminished the amount of one isoform of the peptidyl-prolyl cis-trans isomerase (Cpr3), the heat shock protein Hsp70, two ribosomal proteins (Rpl20B and Yst1) and the peroxiredoxin Tsa1, while increased the amount of the phosphoprotein Tkl1. The translational elongation factor Cef3 is a unique member of this group, since the normalized spot volume of its nonphosphorylated (spot 44) and phosphorylated (spot 27) forms were drastically reduced in the Mut samples.

In Group 3 we placed 11 phosphoproteins whose protein content was not affected significantly, but their phosphorylation changed in the knock out strain (Table 1). The phosphorylation sensitive Pro-Q Diamond staining intensity was reduced in 7 of these proteins (Aip2, Cyp5, Guk1, Ham1, Rpl9B, Rps7A and Tif1) and was enhanced in 4 of them (Eft2, Dug1-like, Rad23 and Rpp0) to a statistically significant level. To support our data the original gel images are shown for Eft2 in S3A Fig, as a typical example. It should be noted that the phosphorylation of the acidic ribosomal protein Rpp0 increased in both spots 20 and 21. A pI shift between the two spots (S3B and S3C Fig) indicates a post-translational modification leading to a more acidic pI of the protein present in spot 21 as compared to the content of spot 20. This can be explained by the differential phosphorylation states of the protein that can be phosphorylated at Ser302 and Thr205 by casein kinase 2 with high and medium probability, respectively, while Thr292 can be phosphorylated either by DNA-dependent protein kinase or casein kinase 2 with the same medium level of probability according to Motif scan analysis.

The two members of Group 4 were identified in two separate spots (Fig 1) that exhibit different tendencies in phosphorylation (Table 1). The deoxyhypusine hydroxylase Lia1-like was found in spots 17 and 18 with the same MW (S3D and S3E Fig) indicating the presence of two different post-translationally modified protein forms, so-called proteoforms. Concerning the more acidic proteoform (spot 18) the apparent decrease in phosphorylation resulted from the decrease in the protein amount, while in the proteoform in spot 17 a decrease in the phosphorylation without any significant change in the protein level was observed. It is likely that this protein has multiple sites for phosphorylation as Motif scan suggests that Tyr85 is a strong Abl kinase site, while Ser12 and Ser178 can be phosphorylated by the DNA-dependent and AMP-dependent protein kinases, respectively, with a medium level of probability. As mentioned before, phosphorylated ubiquitin-activating enzyme Uba1 was identified in spots 30 and 31 (S3F and S3G Fig). The phosphorylation increased significantly in spot 30 in correlation with the increase in the RuBPS protein staining, while in spot 31 the phosphorylation level decreased but the protein level remained unchanged. The pI shift observed between spots 30 and 31 indicates different phosphorylation states of the same protein; suggesting that a hyperphosphorylated form is present in spot 30 and a hypophosphorylated form is in spot 31. In correlation with this explanation a Motif scan search indicated three strong phosphorylation sites in the Uba1 protein: Tyr245, Ser587, and Ser987 can be phosphorylated by Src kinase, DNA-dependent protein kinase, and PKC mu isoform with high probability. Taken together our data revealed that the absence of CaPpz1 decreased the phosphorylation of one specific form of Lia1-like and Uba1 enzymes.

In summary, we found that the protein level of 9 proteins (including 7 phosphoproteins) decreased and 5 proteins (including 2 phosphoproteins) showed an opposite tendency in the *cappz1* mutant *C*. *albicans*. We tested the expression of the genes coding for all of the investigated proteins by quantitative RT-PCR and did not detect significant gene expression changes larger than 1.5 fold upon *CaPPZ1* deletion in any of them (Table 1). Thus, we concluded that the observed changes in protein levels can not be explained by the effect of the CaPpz1 phosphatase on the expression of the selected genes. Rather, it can be attributed to the effect of gene deletion on protein synthesis and protein phosphorylation/dephosphorylation.

### Discussion of the proteomic results

**Fig 2:**
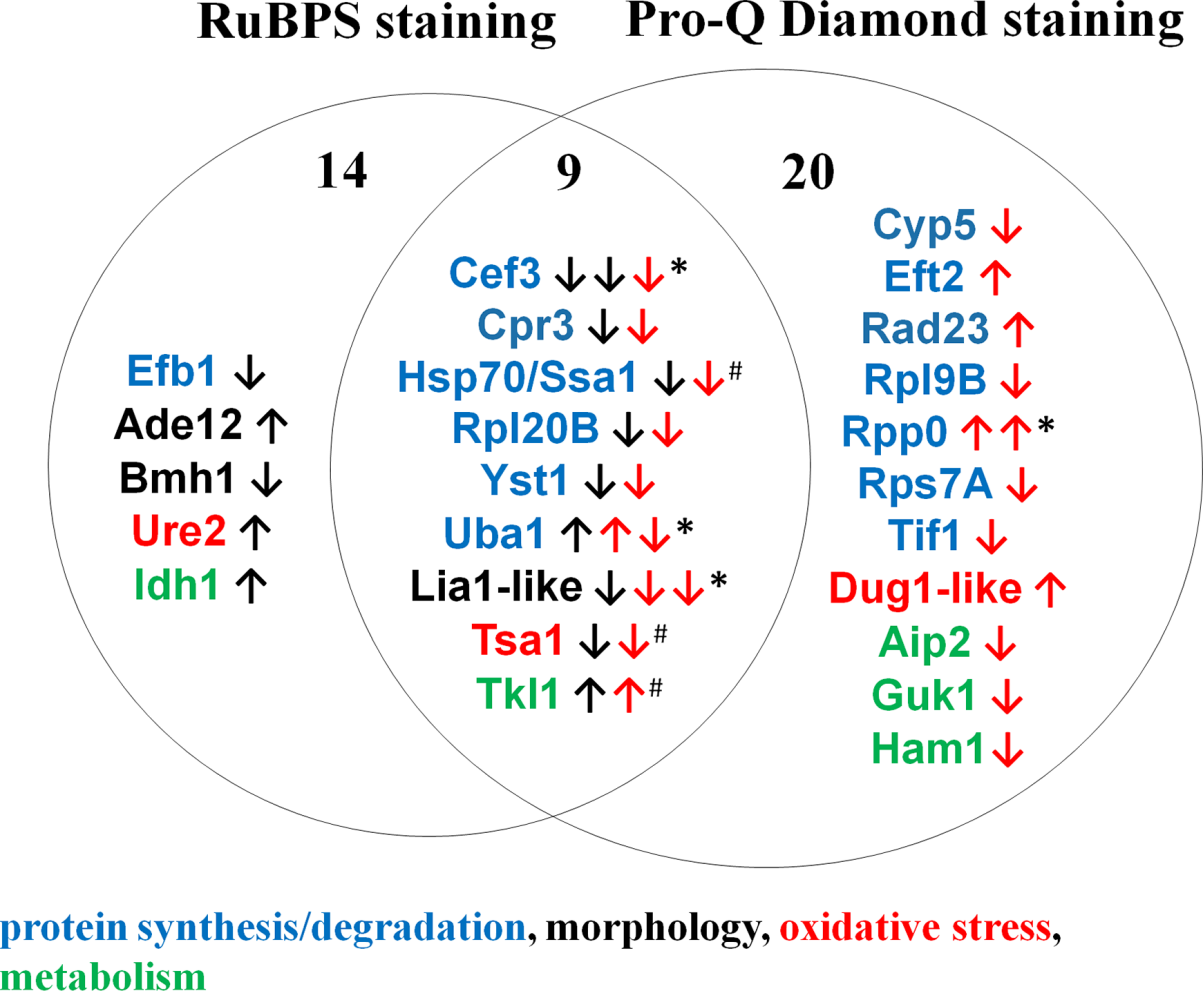
Summary of proteomic results and functional characterisation of the proteins showing significant alterations at protein or phosphorylation level upon *CaPPZ1* deletion. GO functions are indicated by the following color coding: blue: protein synthesis/degradation black: morphology, red: oxidative stress response, green: metabolism. Black and red arrows indicate the directions of changes in RuBPS staining (reflecting protein amounts) and Pro-Q Diamond staining (indicating protein phosphorylation levels), respectively. Numbers indicate the regulated proteins that were stained by either one or both the two dyes. *marks proteins found in two spots (hence the changes are shown by two arrows), and ^#^ labels possible moonlighting proteins.

Based on datamining from two fungal databases the known biochemical and physiological functions of the 25 CaPpz1 affected proteins are listed in Table 2. As many of these functions were deduced by analogy, the names of the better characterised *S*. *cerevisiae* orthologs were also included in the table. According to the GO terms the proteins were grouped in 4 broad functional categories and are presented alongside with the overall proteomic changes in Fig 2. For comparison with Table 1, please, note that Group 1 proteins are in the left side, Group 3 representatives are in the right side; while Group 2 as well as Group 4 members fall in the middle intersecting section of the figure.

**Table 2:** Functional characterization of the *CaPPZ1* deletion affected *C*. *albicans* proteins.

*C*. *albicans* protein^1^	*C*. *albicans* protein name^2^	*S*. *cerevisiae* ortholog^3^	Molecular function^1.3^ / Biological process^1^
***Group 1***			
Ade12	Adenylosuccinate synthetase	Ade12	adenylosuccinate synthase activity, 'de novo' AMP biosynthesis / upregulated in **biofilm**; decreased expression in hyphae vs yeast-form cells
Bmh1	14-3-3 protein homolog	Bmh2	phosphoserine binding, DNA replication origin binding / hyphal growth, Spider **biofilm** repressed
Efb1	Elongation factor 1-beta	Efb1	translation elongation factor activity guanyl-nucleotide exchange factor activity / Spider **biofilm** repressed
Idh1	Isocitrate dehydrogenase [NAD] subunit 1 (mitochondrial)	Idh1	isocitrate dehydrogenase (NAD^+^) activity / soluble protein in hyphae; protein level decrease in stationary phase cultures
Ure2	Protein URE2	Ure2	glutathione peroxidase activity, glutathione transferase activity, phosphoprotein binding, transcription co-repressor activity
***Group 2***			
Cef3	Elongation factor 3	Yef3	translation elongation factor activity, ATPase activity / higher protein amount in stationary phase
Cpr3	Peptidyl-prolyl cis-trans isomerase	Cpr3	peptidyl-prolyl cis-trans isomerase activity / protein folding, apoptotic process, **biofilm** matrix component
Hsp70/Ssa1	Heat shock protein SSA1	Ssa4	chaperon, unfolded protein binding / farnesol-downregulated in **biofilm**, Spider **biofilm** induced
Rpl20B	60S ribosomal protein L20	Rpl20B	structural constituent of ribosome / translation, Spider **biofilm** repressed
Tkl1	Transketolase 1	Tkl1	transketolase activity/pentose phosphate shunt, soluble protein in hyphae, **biofilm** matrix component
Tsa1	Peroxiredoxin TSA1	Tsa1	thioredoxin peroxidase activity, unfolded protein binding / cellular response to oxidative stress, filamentous growth
Yst1	40S ribosomal protein S0	Rps0A	structural constituent of ribosome / translation, Spider **biofilm** repressed
***Group 3***			
Aip2	D-lactate dehydrogenase	Dld2	dehydrogenase activity, actin binding/ flow model **biofilm** repressed
Cyp5	Peptidyl-prolyl cis-trans isomerase	Cpr5	peptidyl-prolyl cis-trans isomerase activity/ protein folding, **biofilm** matrix componenet
Dug1-like	Cys-Gly metallodipeptidase DUG1	Dug1	metallodipeptidase activity, omega peptidase activity/ glutathione catabolism, Spider **biofilm** repressed
Eft2	Elongation factor 2	Eft1	translation elongation factor activity, GTPase activity / higher protein amount in stationary phase
Guk1	Guanylate kinase	Guk1	guanylate kinase activity / **biofilm** matrix component
Ham1	Inosine triphosphate pyrophosphatase	Ham1	deoxyribonucleoside triphosphate pyrophosphohydrolase activity
Rad23	Rad23p	Rad23	damaged DNA binding, contributes to amidase activity, proteasome binding, protein binding, protein bridging, ubiquitin binding
Rpl9B	Likely cytosolic ribosomal protein L9	Rpl9B	structural constituent of ribosome, cytoplasmic translation / protein levels decrease in stationary phase, Spider **biofilm** repressed
Rpp0	60S acidic ribosomal protein P0	Rpp0	structural constituent of ribosome, large ribosomal subunit rRNA binding / cytosolic translation, Spider **biofilm** repressed
Rps7A	40S ribosomal protein S7-A	Rps7A	structural constituent of ribosome / translation, Spider **biofilm** repressed
Tif1	ATP-dependent RNA helicase eIF4A	Tif2	translation initiation factor activity, ATP-dependent RNA helicase activity / Spider **biofilm** repressed
***Group 4***			
Lia1-like	Deoxyhypusine hydroxylase	Lia1	deoxyhypusine monooxygenase activity / filamentous growth, Spider **biofilm** repressed
Uba1	Ubiquitin-activating enzyme E1	Uba1	ubiquitin activating enzyme activity/ protein levels decrease in stationary phase yeast cultures

Based on

^1^http://www.candidagenome.org/

^2^http://www.uniprot.org/, and

^3^http://www.yeastgenome.org/. Biofilm related functions are highlighted in bold face.

According to their functions 12 out of the 25 proteins (including 1 transcriptional initiation factor, 3 translational elongation factors, 5 ribosomal components and 3 chaperons) are involved in protein synthesis and 2 of them are related to proteasomal protein degradation. The left and middle sections of Fig 2. show, that the level of 7 proteins that are related to protein synthesis decreases, and the level of the ubiquitin-activating enzyme E1 (Uba1) increases in the knock out strain. This suggests that the deletion of *CaPPZ1* has a negative effect on protein synthesis in the mutant. The role of *S*. *cerevisiae* Ppz1 in translation has been reported previously [31] in association with the regulation of nonsense suppression efficiency. The phosphorylation of the proteins in this functional category was affected in a more diverse manner in the deletion mutant samples; the level of phosphorylation increased in Eft2, Rad32 and Rpp0 (in two separate forms of the latter) and decreased in ribosomal proteins Rpl6B and Rps7A, as well as in the transcription initiation factor Tif1 and one of the proteoforms of the ubiquitin ligase Uba1. The data suggest that translational elongation factor-2, the up till now uncharacterised Rad32 protein and 60S acidic ribosomal proteins may be direct substrates of CaPpz1 phosphatase. In comparison with *S*. *cerevisiae*, where the EF1Bα/Efb1 translational elongation factor was reported to be a Ppz1 substrate/target [13], we found that the amount of the *C*. *albicans* ortholog of the Efb1 was significantly reduced in the phosphatase mutant, but the phosphorylated form of this protein we were not able to detect by the Pro-Q Diamond staining (Table 1). We identified the phosphorylated form of elongation factor 3 (Cef3) and detected decreasing protein (in spots 27 and 44) as well as phosphorylation levels (in spot 27) upon the phosphatase deletion (Table 1). On the other hand, we noted a significant enhancement of the phosphorylation of elongation factor 2 (Eft2) under the same conditions suggesting that the latter factor is the target of CaPpz1 in *C albicans* (S3A Fig). It is important to note that Eft2 was shown to be a direct target of sordarins, the antifungal agents that inhibit protein synthesis [32]. The functional equivalence between the orthologous *C*. *albicans* Eft2 and *S*. *cerevisiae* Efb1/Efb2 elongation factors was proven by the fact, that Eft2 complemented the *efb1 efb2* double mutant [33]. Our Motif scan analysis identified Tyr175 as a preferred site for Lck kinase and revealed that Thr713, Thr404, as well as Ser579 can be phosphorylated by the second messenger regulated PKA and PKC, as well as by the cell cycle associated Cdk2 or Cdk5 protein kinases, respectively. As it was mentioned in the previous section from a similar motif search, we know that the ribosomal protein Rpp0 exhibits several casein kinase 2 consensus sites. It was reported, that the phosphorylation of the budding yeast Rpp0 ortholog by casein kinase is not essential for translation but may regulate the expression of some genes that are involved in osmoregulation [34]. Since both Rpp0 and Eft2 are related to translation, it is possible that CaPpz1 could regulate protein synthesis *via* these two targets. Unfortunately, the Rad32-like protein has not been characterized yet, so the significance of its phosphorylation can not be predicted at the moment.

In agreement with the role of CaPpz1 in the oxidative stress response of the fungus we detected significant changes in three oxidative stress related proteins in the Mut samples (Fig 2). Ure2 has glutathione peroxidase activity, the Dug1-like Cys-Gly metallopeptidase takes part in glutathione metabolism and Tsa1 exhibits thioredoxin peroxidase activity (Table 2).

In addition, we identified 5 metabolic enzymes that were affected by the deletion of the CaPpz1 phosphatase (Fig 2). The amounts of the mitochondrial isocitrate dehydrogenase Idh1 and the cytosolic transketolase Tkl1 increased in the knockout cells (Table 1). Meanwhile, the phosphorylation of the uncharacterized dehydrogenase Aip1, guanylate kinase Guk1 and inosine triphosphate pyrophosphatase Ham1 decreased in the mutant (Table 1). These and other cases of protein dephosphorylation effects in the phosphatase mutant are most probably related to the interplay between PPZ and the MAP-kinase pathway [18] as well as by the opposite genetic interactions between PPZ and the Sit4 protein phosphatases [35] in fungi. Concerning the changes in common metabolic enzymes it should be noted, that according to a recent hypothesis several common enzymes may play additional moonlighting functions. For example in the pathogenic fungi *bona fide* cytosolic enzymes, ribosomal proteins and chaperons may acquire novel functions after transportation to the cell surface [36]. From this point of view three of the proteins identified in the present study Hsp70/Ssa1, Tsa1 and Tkl1 have been detected on the cell surface of *C*. *albicans* by proteomic methods [37,38].

Finally, in the Mut strain we found a significant change in the levels of 3 proteins that were previously reported to be involved in the morphological transition of *C*. *albicans* (Table 2). The amount of Ade12 adenylosuccinate synthase, an enzyme that is upregulated in biofilm, increased in the absence of CaPpz1, while the amounts of the 14-3-3 scaffolding protein homolog Bmh1 and that of the Lia1-like deoxyhypusine hydroxylase decreased in the mutant in correlation with the fact that both of them are repressed in Spider biofilm [25]. These observations revealed the possibility of a functional role for the phosphatase in biofilm formation. After noting a coincidence between morphology related proteins and biofilm we carried out a careful database search and found that out of the 25 proteins examined 17 were biofilm matrix components or their genes showed altered expression levels during biofilm formation (Table 2). The proteins Cpr3, Cyp5, Guk1 and Tkl1 were localized to the biofilm matrix. According to Table 1 the amount of Tkl1 was elevated, and the amount of Cpr3 diminished in the phosphatase knockout strain, while the Cyp5 and Guk1 proteins displayed decreased phosphorylation levels without significant changes in their protein amounts. The original study that identified these four proteins as biofilm matrix components [39] concluded that in general terms the *Candida* strains with normal *versus* impaired biofilm forming capabilities had an almost identical extracellular matrix proteome. However, the phosphorylation state of the biofilm forming proteins has not been investigated before. In addition, the transcript levels of 13 CaPpz1 affected proteins identified in this study (Table 2) were reported to change during biofilm formation [25].

### Effects of *CaPPZ1* deletion on biofilm formation

Although, under our experimental conditions we did not observe a significant change in the expression of the corresponding genes (Table 1), the coincidences between our proteomic results and the localization or expression of biofilm related proteins (Table 2) prompted us to investigate if CaPpz1 was indeed involved in biofilm formation. For this purposes we compared the biofilm forming capability of the Ctrl and Mut strains under different conditions. First we conducted experiments in RPMI-1640, that has been used in most of the biofilm studies, because it promotes an almost uniform filamentous growth. In this medium both strains showed excessive biofilm production and very small or negligible amount of planktonic cells. Biofilm accounted for the majority of total biomass after incubation in RPMI. The total dry biomass of Mut was significantly higher, but biofilm to total biomass ratio of the Ctrl only slightly exceeded that of the knockout strain. Crystal violet (CV) staining did not show any significant differences between the two strains' biofilm formation either (Table 3). Next, we carried out the same tests in the Spider medium, which was used in one of the key studies in the field [25]. Under this condition the extent of biofilm formation was much lower for both strains in comparison to the RPMI medium (Table 3). In contrast with the previous results, in the Spider medium the knockout strain showed significantly drier biofilm mass; the mean biomass per well value was approximately 4 times higher in the Mut than in the Ctrl strain. The better biofilm forming capability of the knockout strain in this medium was demonstrated the by biofilm to total biomass ratio as well as by CV staining (Table 3). The differences between biofilms of a given strain in different media are well documented. In a recent publication [40] it was reported that *C*. *albicans* in contrast to RPMI in Spider medium did not form a uniform yeast cell polylayer at the substrate. Also in Spider medium, hyphae did not display the high degree of vertical orientation they did in biofilms formed in RPMI medium. Note that this observation was made with strains of a/α mating type, and the strains used in this study have the same mating type. Thus, apparently the specific differences in the architecture of Spider biofilms magnified the differences between the control and KO strains.

**Table 3:** Effect of *CaPPZ1* deletion on the biofilm formation. Differences between the control QMY23 (Ctrl) and *cappz1* knockout mutant (Mut) strain's ability to form biofilms in RPMI-1640 and Spider medium were assessed by determining total biomass, biofilm mass to total biomass ratio, and by crystal violet (CV) staining of biofilms. n = 36 for each condition, and each strain, in three independent experiments.

	Biofilm Biomass (mg)				Biofilm/Total Biomass ratio				CV staining (A 595 nm)			
Medium	RPMI-1640		Spider medium		RPMI-1640		Spider medium		RPMI-1640		Spider medium	
Strain	Ctrl	Mut	Ctrl	Mut	Ctrl	Mut	Ctrl	Mut	Ctrl	Mut	Ctrl	Mut
Mean	6.30	7.60	0.30	1.20	0.97	0.94	0.12	0.24	0.25	0.23	0.06	0.14
SD	1.39	1.80	0.23	0.52	0.04	0.05	0.09	0.15	0.04	0.03	0.02	0.04
Significance	p < 0.001		p < 0.001		p < 0.042		p < 0.001		p < 0.071		p < 0.001	

Microscopic examination of biofilms formed in 12-wells showed excessive hyphal growth for both strains in RPMI medium with few detectable yeast cells (S4A and S4B Fig). In this medium, blastospore formation was more pronounced in the case of the knockout strain (S4B Fig). In Spider medium, yeast cells in the biofilm were more abundant among the hyphae in both strains (S4C and S4D Fig). However, the Ctrl strain merely formed small, isolated patches of adherent biofilm in this medium (fragile biofilm sensu [41]), whereas the Mut strain's biofilm formation was more pronounced, with biofilms covering larger areas of the plastic wells. Our qualitative observations cannot be supported by quantitative data as statistical image analysis was prevented by the technical constraints of the phase contrast imaging applied to thick biofilms. Despite this limitation we can state that the CaPpz knockout strain showed better biofilm forming capability in the selective Spider medium than the corresponding Ctrl strain indicating that Cappz1 has a direct or indirect role in the biofilm formation of *C*. *albicans*.

## Conclusions

**Fig 3:**
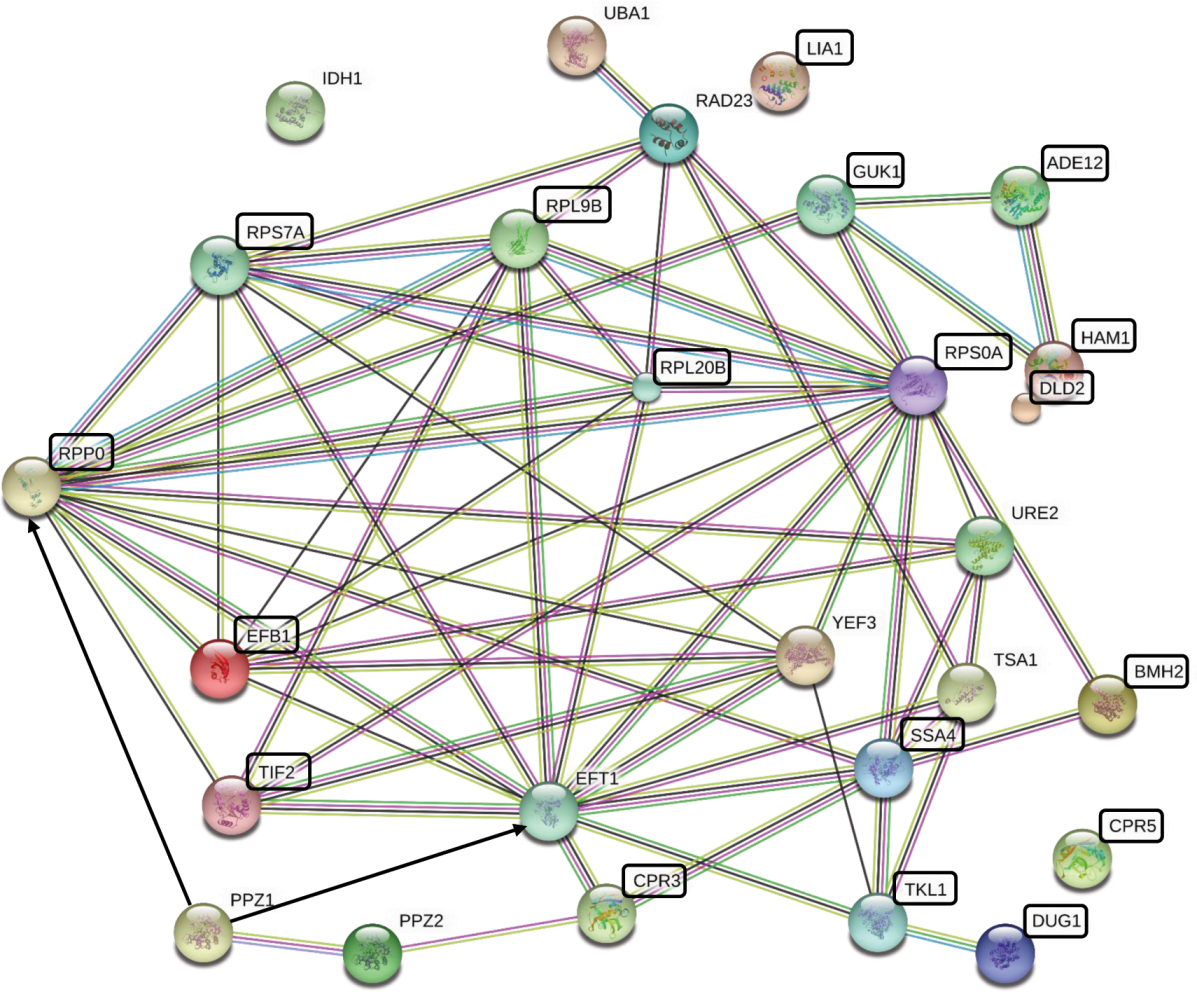
Protein-protein interaction network of the *S*. *cerevisiae* orthologs of the *C*. *albicans* proteins affected by CaPpz1 deletion. The network of proteins listed in Table 3 and the two budding yeast phosphatase paralogs Ppz1 and Ppz2 was generated using String 10.5 with default settings at medium stringency. The network nodes are proteins identified by the gene name, while the lines are edges representing functional associations based on different types of evidence according to the String color coding scheme. The black boxes highlight the proteins which are associated with biofilm formation. The black arrows represent the two putative substrates of Ppz1.

Our proteomic and phosphoproteomic investigations revealed that the deletion of the fungus-specific CaPpz1 from *C*. *albicans* affected the amount and phosphorylation of proteins involved in protein synthesis/degradation, morphological transitions, oxidative stress response and metabolism. We identified two putative substrates of the phosphatase Eft2 and Rpp0 that are critical regulators of translational elongation. These proteins are interlocked according to their functions and localizations (Table 2). The interactome of *C*. *albicans* proteins has not been worked out yet, but the interrelationships of these proteins can be anticipated by the analysis of their *S*. *cerevisiae* orthologs (listed in Table 2) with the aid of the String software. As shown in Fig 3. the five ribosomal proteins (Rpp0, Rps7A, Rpl9B, Rpl20B, and Rps0A) form a strongly associated cluster that interacts extensively with the four translation factors (Efb1, Tif2, Eft1, and Yef3) and connects to the ubiquitin activating enzyme Uba1 *via* Rad23, as well as to the triade of the nucleotide metabolising enzymes (Guk1, Ade12, and Ham1) *via* Guk1. The chaperon (Ssa4, Tsa1, and Crp3) and scaffold (Bmh2 and Ure2) proteins interact with the members of either the ribosomal protein cluster or the translation factor cluster or with both of them. Dug1 metalloproteinase connects to the main block through the transketolase Tlk1. Many of the metabolic enzymes like Idh1, Lia1, and Dld2 are outliers, indicating that they are most likely indirectly related to the rest *via* the altered metabolic fluxes in the mutant cells. According to the String database the Ppz2 phosphatase isoenzyme connects to the translation factor cluster *via* the peptidyl-prolyl cis-trans isomerase Cpr3, while the other isomerase isoform Cpr5 remains isolated. Based on our results with the *C*. *albicans* proteins we suggest that the Ppz1 phosphatase dephosphorylates two of the hub proteins of the cluster, namely the orthologs of the translation elongation factor Eft1 and the ribosomal protein Rpp0, which are also connected to each other. This hypothesis could explain the effects of protein phosphatase Z on protein synthesis and also would rationalize its effects on the associated proteins. Due to the complicated genetic manipulation of *C*. *albicans*, it would be more realistic to test our suggestion in *S*. *cerevisiae*.

Our observations are in good agreement with the results of previous genetic studies in terms of PPZ functions in the *C*. *albicans* and the much better characterised budding yeast *S*. *cerevisiae*. In addition, we found a strong correlation between the proteomic consequences of CaPPZ1 gene deletion and biofilm formation. We tested this assumption experimentally and found that in the selective Spider medium the knock out mutant showed more pronounced biofilm formation than the genetically matching control strain. Thus, by the application of the unbiased proteomic approach we identified a novel function for the phosphatase in *C*. *albicans*. This is an important observation, since the adhesion of the fungi to plastic surfaces can result in the contamination of medical equipment and the tight packing of the biofilm may prevent the eradication of the fungal pathogen by conventional antifungal agents that cannot reach the protected cells in the deep layer of the film [42,43]. Fig 3 shows that the orthologs of most of the biofilm related proteins are associated with the network of the potential phosphatase regulated proteins. They include all of the five ribosomal proteins, two of the translation factors, as well as many chaperon and scaffolding proteins suggesting a connection between protein synthesis and biofilm formation. In addition, several metabolic enzymes, either associated to the main cluster (Guk1, Ade12, Ham1, and Tkl1) or isolated outliners (Lia1 and Dld2) may be involved in biofilm deposition through their metabolic activities or moonlighting functions. The reasons behind this associations is presently unknown, but our observation illustrates well how proteome data can be used to identify previously not researched processes, such as the role of CaPpz1 in biofilm formation.

## Supporting information

S1 Fig Growth curves for the *C*. *albicans* strains. The genetically matching control QMY23 (●) and the *cappz1* phosphatase deletion mutant (o) strains were cultivated under identical conditions as described in the Materials and methods. The optical density (OD) of the samples was monitored at 640 nm wavelength in three parallel experiments. The mean and standard deviation of a representative preparation are shown. Similar results were obtained in five independent preparations, confirming that the growth rate of the mutant strain is somewhat reduced in comparison to that of the control strain.
(TIF)

S2 Fig Testing oligonucleotide primers. All of the primer pairs that were designed for RT-qPCR were tested on QMY23 cDNA target by PCR with Phusion DNA polymerase (Thermo Scientific). The temperature profile started with a single denaturation step for 2 min at 95°C that was followed by 30 cycles of 30 s at 95°C, 30 s at 60°C, and 10 s at 72°C, and was concluded with a polishing step at 72°C for 10 min. The amplicons were separated in 1% agarose by gel electrophoresis and were stained with GelRed (Biotium). The sizes of the DNA standards (St.) are given in base pairs. The properties of the primers as well as the sizes of the expected PCR products are summarized in S1 Table. The figure demonstrates that all of the gene specific primer pairs produce a single DNA band of the calculated size under the simulated qPCR conditions.
(TIF)

S3 Fig Magnified image of the three gel regions containing the highlighted spots of selected proteins. Original gel sections are labelled as C1-3 for Ctrl and M1-3 for Mut samples after RuBPS protein and Pro-Q Diamond phosphoprotein staining. Spot numbers (see Table 1 and S2 Table) and the corresponding protein names (in parenthesis) are listed on the right side of each panel. All of the original data and their individual densitometric evaluation can be found at http://bmbi.med.unideb.hu/en/proteomics-core-facility.
(TIF)

S4 Fig Microscopic morphology of the biofilms. Examples of biofilms formed by the QMY23 control (Ctrl) and *cappz1* mutant (Mut) strains after 24 hours incubation at 37°C either in RPMI-1640 or in Spider medium. a: Ctrl in RPMI medium. b: Mut in RPMI. c: Ctrl in Spider medium. d: Mut in Spider medium. Scale bar: 100 μm.
(TIF)

S1 Table Oligonucleotide primers used for RT-qPCR analysis. Oligonucleotide sequences are given in 5’ to 3’ direction. The calculated Tm points (in the order of upper to lower primers) and the expected amplicon sizes are also given for each primer pair.
(PDF)

S2 Table Identification of *C*. *albicans* proteins by MS/MS. The peptide sequences identified in all of the analyzed protein spots are listed with the protein name, the Uniprot accession number and the protein identification details in the format characteristic for the search engine used. All MS/MS spectra were analyzed by ProteinPilot, and in those cases where the analysis did not give sufficient data for protein identification a Mascot search was also performed.
(XLSX)

S3 Table Identification of proteins in spots that exhibit significant Pro-Q Diamond staining intensity changes in the absence of CaPpz1. (PDF)

S4 Table Identification of proteins in spots that exhibit significant RuBPS staining intensity changes in the absence of CaPpz1. (PDF)
