# Supplementary figures and images for: Proteomic analysis of protein phosphatase Z1 from Candida albicans
Source: PLoS One. 2017 Aug 24;12(8):e0183176. doi: 10.1371/journal.pone.0183176 (PMC5570430; doi:10.1371/journal.pone.0183176)

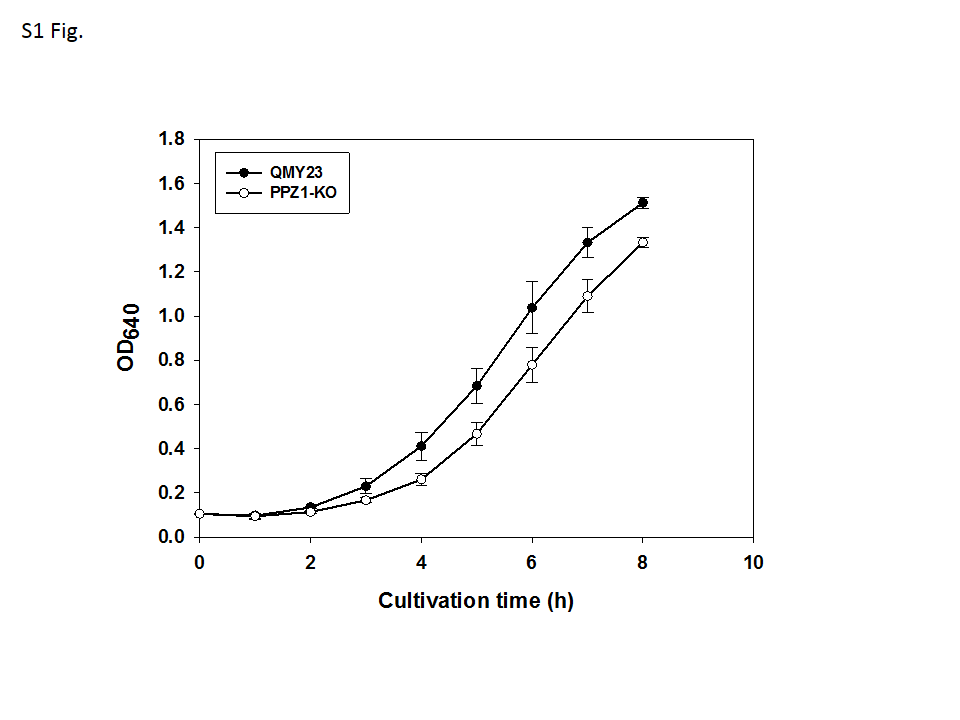

Supplement: S1 Fig — The genetically matching control QMY23 (●) and the cappz1 phosphatase deletion mutant (o) strains were cultivated under identical conditions as described in the Materials and methods. The optical density (OD) of the samples was monitored at 640 nm wavelength in three parallel experiments. The mean and standard deviation of a representative preparation are shown. Similar results were obtained in five independent preparations, confirming that the growth rate of the mutant strain is somewhat reduced in comparison to that of the control strain. (TIF) [file pone.0183176.s001.tif]

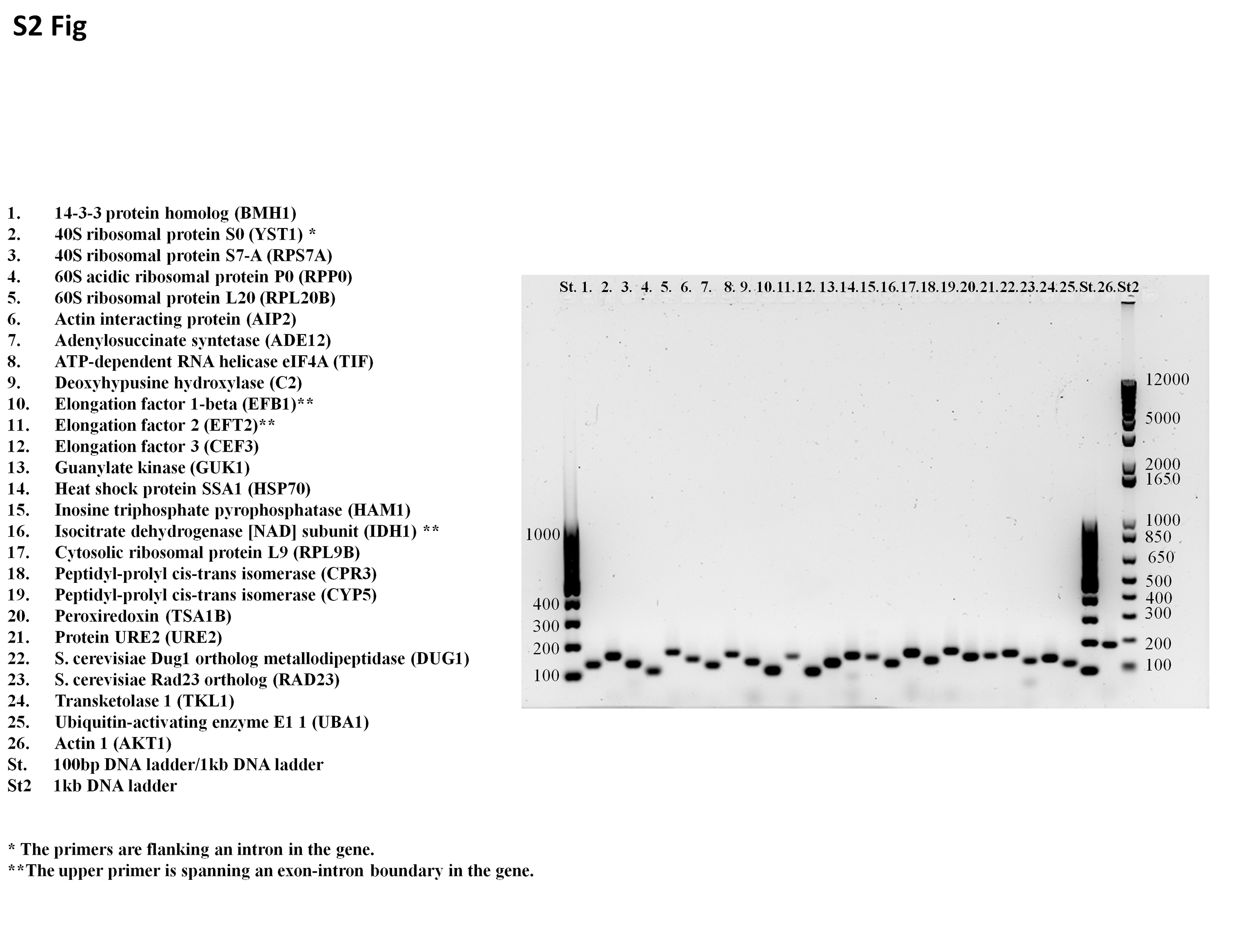

Supplement: S2 Fig — All of the primer pairs that were designed for RT-qPCR were tested on QMY23 cDNA target by PCR with Phusion DNA polymerase (Thermo Scientific). The temperature profile started with a single denaturation step for 2 min at 95°C that was followed by 30 cycles of 30 s at 95°C, 30 s at 60°C, and 10 s at 72°C, and was concluded with a polishing step at 72°C for 10 min. The amplicons were separated in 1% agarose by gel electrophoresis and were stained with GelRed (Biotium). The sizes of the DNA standards (St.) are given in base pairs. The properties of the primers as well as the sizes of the expected PCR products are summarized in S1 Table. The figure demonstrates that all of the gene specific primer pairs produce a single DNA band of the calculated size under the simulated qPCR conditions. (TIF) [file pone.0183176.s002.tif]

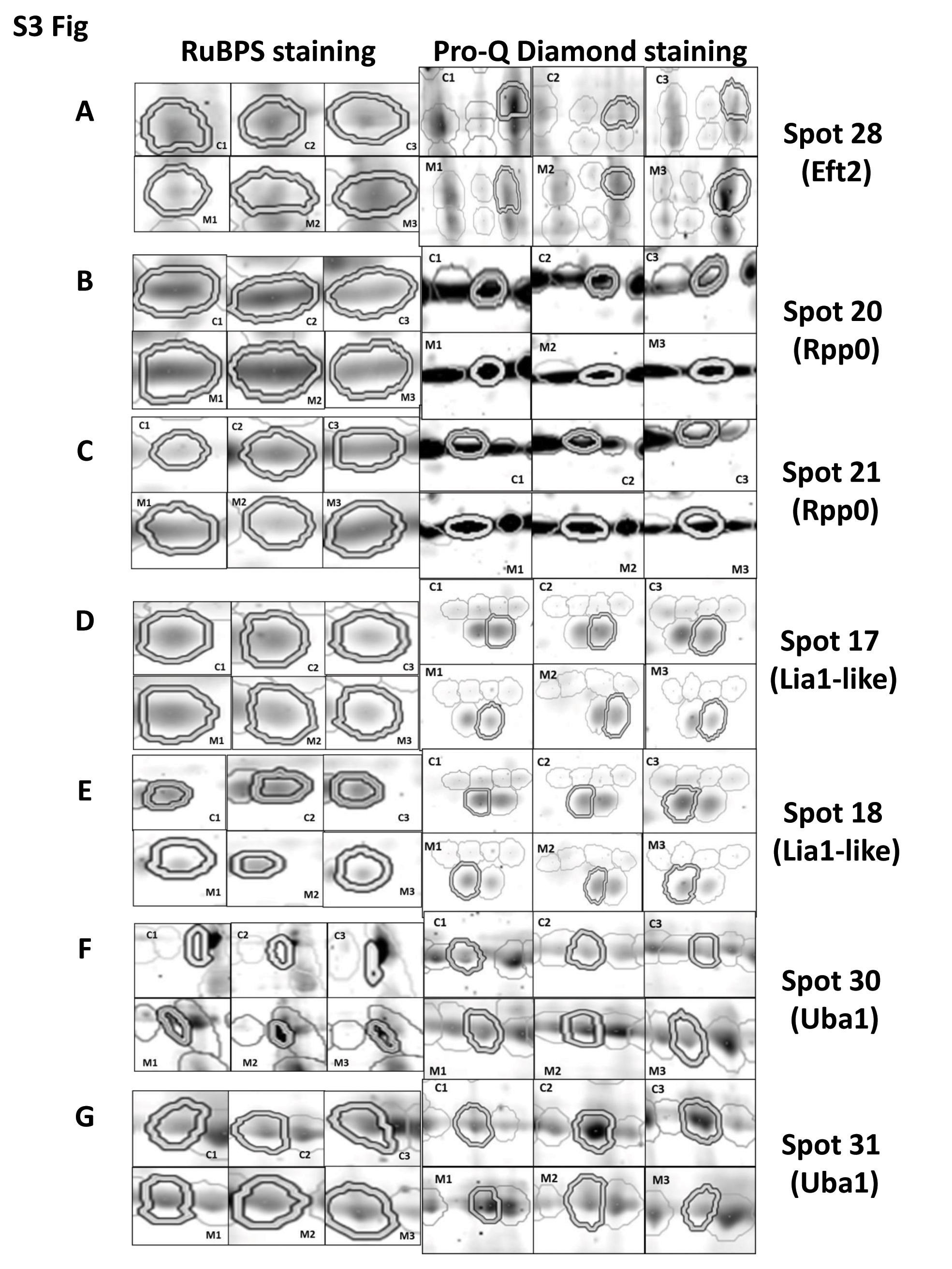

Supplement: S3 Fig — Original gel sections are labelled as C1-3 for Ctrl and M1-3 for Mut samples after RuBPS protein and Pro-Q Diamond phosphoprotein staining. Spot numbers (see Table 1 and S2 Table) and the corresponding protein names (in parenthesis) are listed on the right side of each panel. All of the original data and their individual densitometric evaluation can be found at http://bmbi.med.unideb.hu/en/proteomics-core-facility. (TIF) [file pone.0183176.s003.tif]

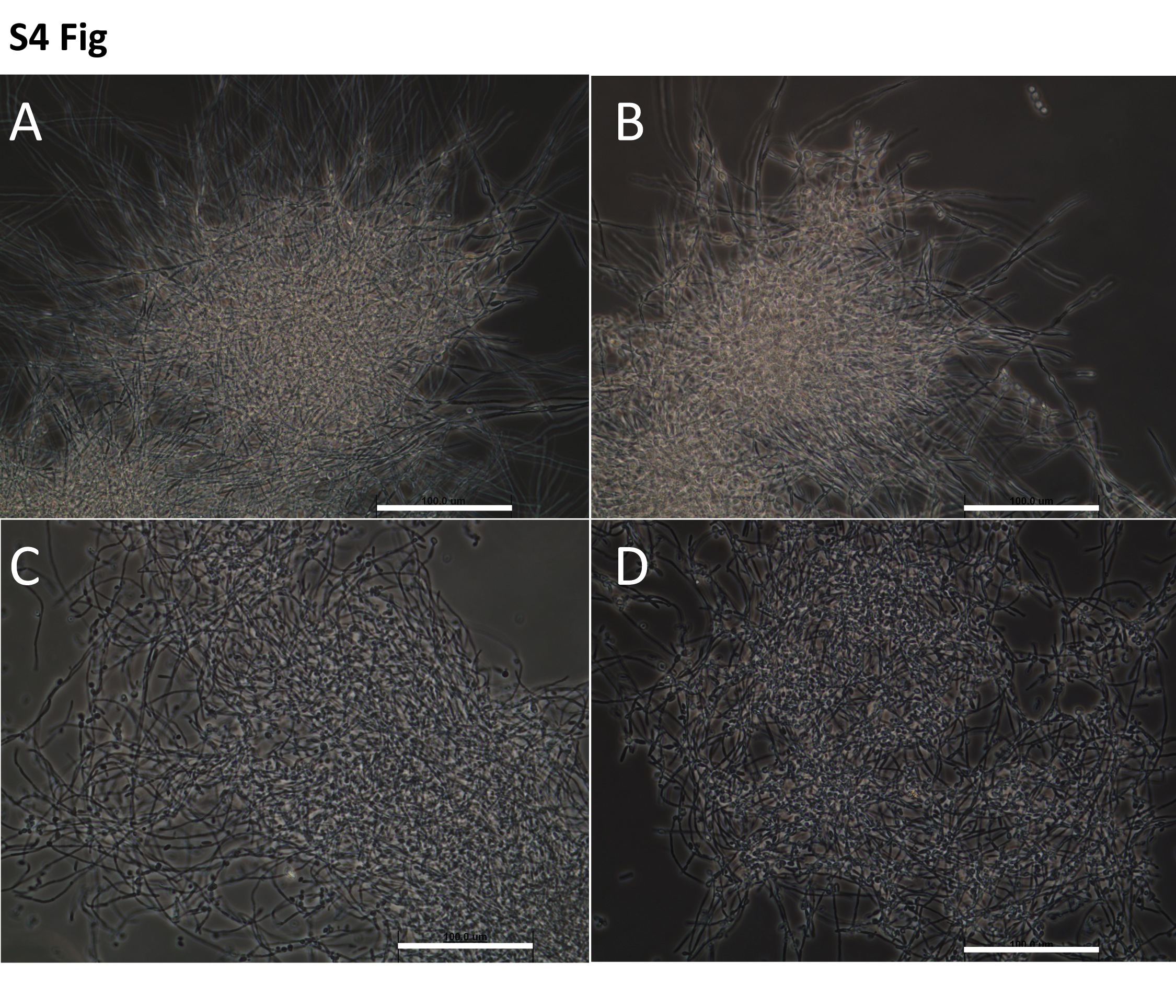

Supplement: S4 Fig — Examples of biofilms formed by the QMY23 control (Ctrl) and cappz1 mutant (Mut) strains after 24 hours incubation at 37°C either in RPMI-1640 or in Spider medium. a: Ctrl in RPMI medium. b: Mut in RPMI. c: Ctrl in Spider medium. d: Mut in Spider medium. Scale bar: 100 μm. (TIF) [file pone.0183176.s004.tif]
