# Supplementary material for: Proteomic analysis of protein phosphatase Z1 from Candida albicans
Source: PLoS One. 2017 Aug 24;12(8):e0183176. doi: 10.1371/journal.pone.0183176 (PMC5570430; doi:10.1371/journal.pone.0183176)
Supplement: S1 Table — Oligonucleotide sequences are given in 5’ to 3’ direction. The calculated Tm points (in the order of upper to lower primers) and the expected amplicon sizes are also given for each primer pair. (PDF) [file pone.0183176.s005.pdf]

S1 Table. Oligonucleotide primers used for RT-qPCR analysis

Protein name[1]	Gene ID[2]	Upper (U) primer (5'-3')	Lower (L) primer (5'-3')	Tm U - L (C°)	Product (bp)
14-3-3 protein homolog	*C1_03220C*	CACCAACCCATCCAATCAG	AATCAGCAACAGCATCATCG	62.2-62.2	126
40S ribosomal protein S0	*C3_05370C**	TGTTATTAGCTGCCAACGTCC	CAGCAGCAATGATTCTGGC	62.3-63.0	153
40S ribosomal protein S7-A	*C3_01490W*	TCAAGCTTTCGTTGATTTGG	GGTGGTGGAACGAAAACAG	61.2-61.4	129
60S acidic ribosomal protein P0	*C7_00990W*	TCCGAAGCTACCTTGTTGAAC	GATGGATGATGGGAAGACTTG	60.5-58.5	103
60S ribosomal protein L20	*C4_01520C*	GTTGGTGCTGTTGAAACCATG	CTCTGTGTGGTAATGGGAATTTC	61.6-61.8	169
Adenylosuccinate synthetase	*C1_09640W*	TTGGTTGAAGGTGCTAATGC	ATGGTTCTTGGAGGAATACCC	61.0-61.3	122
ATP-dependent RNA helicase eIF4A	*C1_01350C*	ACCGCTACCTTTACCATTTCTG	ACCACCAATAGAAGCATGGAC	61.6-61.2	165
Cys-Gly metallodipeptidase DUG1	*C5_04300C*	GATATGGTTGCTCCTTTGACTG	ATGAAAGGGATGGGAATCTC	60.9-60.1	155
Deoxyhypusine hydroxylase	*C2_07290W*	CGAAGCTCTTGGTGCTCTTG	GCTTTTTCTGAGTTTTCCCAGTG	63.6-63.5	138
D-lactate dehydrogenase	*C3_03040W*	CGGTGACTTCCCATTCTATG	ATTTGAGCCTCATCTTGTGC	60.3-60.2	144
Elongation factor 1-beta	*C4_04480C***	ATGGTACTACTGCCACTCAAGC	CAGTGAATGAAGCAATGTGGT	60.6-61.2	102
Elongation factor 2	*C2_03100W***	ACAAGATGGTTGCTTTCACTATTG	TTGGCAGCGGAAATAATACC	61.6-62.1	154
Elongation factor 3	*C5_01580C*	GATCACAATTGGGTCCAAGG	AGCAGCGGCAATCTTGTTAC	62.5-62.6	105
Guanylate kinase	*C5_03790W*	CATCTGCCAATAGACCAATCG	TGGTTTTCTGGTGGTGTTTG	62.7-61.8	128
Heat shock protein SSA1	*C1_13480W*	CATTAGTGGTGCTTATGGTGC	ACTTCTTCAACAGTTGGTCCA	59.5-60.0	164

Inosine triphosphate pyrophosphatase	*C5_03860W*	GACTCCTCCTCCTCCTCATC	CAATGACTTTAGCAGCAGCTTG	60.2-62.3	148
Isocitrate dehydrogenase [NAD] subunit 1 (mitochondrial)	*C1_09630W***	CGTTATTGCTCAAAAGAACTCC	CTTGACCAGCACCATCACC	60.1-62.7	125
Peptidyl-prolyl cis-trans isomerase	*C2_02320C*	TGCTTCAAGGTGGTGATTTTG	GGACCAGCATTGGCCATAG	63.3-63.8	139
Peptidyl-prolyl cis-trans isomerase	*C3_06360C*	CAATCGCATTGATTGCTTC	ACAACCGTGCCAAAGAGAC	60.2-61.1	170
Peroxiredoxin TSA1	*C3_06180C*	AAATTTGCCGAAAAGGATGC	TCTGGACAAGGAGTGGTTGG	62.2-61.6	150
Protein phosphatase Z1	*CR_06420W*	TCACAACAACGTCCACAACCA	GACAACGGTGAACCTGGC	62.9-61.1	173
Protein URE2	*C2_04710C*	GGTGAATGTTCATTATGGTCG	CTTTCTACAGCACTTGGTACTGG	60.1-60.2	152
Rad23p	*C2_01850W*	AACAACCACAACCAGCAGC	ATGCGGCTCTTAATGCAGC	62.2-63.7	129
Ribosomal 60S subunit protein L9B	*C3_02470C*	CCCAGAAGGTGTCACTGTTG	GCAGCAACGTGTTTTCTGTC	61.5-61.9	165
Transketolase 1	*C1_08320W*	TTCACCTTCGACCAACAATC	GCACCAAATCTGTTCAAACC	60.9-60.4	141
Ubiquitin-activating enzyme E1	*C3_06500W*	CAGGTGAAGAACCATTGAGC	GGCAAACTTGACATAATCGC	60.7-60.5	113
Actin 1	*C1_13700W*	GTTGCTCCAGAAGAACATCC	ACCATCACCAGAATCCAAAAC	59.7-61.0	192

Based on [1]http://www.uniprot.org/ and [2] http://www.candidagenome.org/.
* The primers are flanking an intron in the gene.
**The upper primer is spanning an exon-intron boundary in the gene.
C1_13700W was used as an internal control in all experiments.
