# Supplementary material for: Proteomic analysis of protein phosphatase Z1 from Candida albicans
Source: PLoS One. 2017 Aug 24;12(8):e0183176. doi: 10.1371/journal.pone.0183176 (PMC5570430; doi:10.1371/journal.pone.0183176)
Supplement: S3 Table — (PDF) [file pone.0183176.s007.pdf]

S3 Table. Identification of proteins in spots that exhibit significant Pro-Q Diamond-staining intensity changes in the absence of CaPpz1

Protein Name[1]	Protein ID[1]	Systemic gene name[2]	Standard gene name[1]	Protein abbreviation[2]	Spot ID	Fold change	p value
40S ribosomal protein S0	O42817	*C3_05370C*	*YST1*	Yst1	22	-2.79	0.041
40S ribosomal protein S7-A	Q5AJ93	*C3_01490W*	*RPS7A*	Rps7A	04	-2.58	0.047
60S acidic ribosomal protein P0*	A0A1D8PQS0	*C7_00990W*	*RPP0*	Rpp0	20	2.56	0.046
					21	2.63	0.005
60S ribosomal protein L20	A0A1D8PLC9	*C4_01520C*	*RPL20B*	Rpl20B	10	-4.81	0.019
ATP-dependent RNA helicase eIF4A	P87206	*C1_01350C*	*TIF1*	Tif1	23	-2.05	0.014
Cys-Gly metallodipeptidase DUG1	Q5AKA5	*C5_04300C*	*DUG1*	Dug1-like**	36	1.37	0.008
Deoxyhypusine hydroxylase*	Q59Z14	*C2_07290W*	*LIA1*	Lia1-like**	17	-3.09	0.019
					18	-2.32	0.008
D-lactate dehydrogenase	A0A1D8PJK5	*C3_03040W*	*AIP2*	Aip1	34	-2.05	0.026
Elongation factor 2	Q5A0M4	*C2_03100W*	*EFT2*	Eft2	28	2.79	0.008
Elongation factor 3	P25997	*C5_01580C*	*CEF3*	Cef3	27	-2.38	0.008
Guanylate kinase	A0A1D8PNS0	*C5_03790W*	*GUK1*	Guk1	03	-4.57	0.003
Heat shock protein SSA1	P41797	*C1_13480W*	*HSP70*	Hsp70/Ssa1	32	-3.32	0.024
Inosine triphosphate pyrophosphatase	Q59N80	*C5_03860W*	*HAM1*	Ham1	01	-2.79	0.034
Peptidyl-prolyl cis-trans isomerase	Q5ALM6	*C2_02320C*	*CPR3*	Cpr3	02	-2.59	0.018
Peptidyl-prolyl cis-trans isomerase	A0A1D8PKL0	*C3_06360C*	*CYP5*	Cyp5	07	-2.02	0.0003
Peroxiredoxin TSA1	Q9Y7F0	*C3_06180C*	*TSA1*	Tsa1	16	-2.18	0.028
Rad23p	A0A1D8PGE3	*C2_01850W*	*RAD23*	Rad23	39	1.95	0.006
Ribosomal 60S subunit protein L9B	Q5AEN2	*C3_02470C*	*RPL9B*	Rpl9B	13	-3.23	0.049
Transketolase 1	Q5A750	*C1_08320W*	*TKL1*	Tkl1	29	1.78	0.045
Ubiquitin-activating enzyme E1*	A0A1D8PKJ3	*C3_06500W*	*UBA1*	Uba1	30	1.90	0.006
					31	-2.31	0.037

Based on [1]http://www.uniprot.org/ and [2] http://www.candidagenome.org/.
*The same protein was identified in two separate spots.
** The abbreviation is based on the name of the *S. cerevisiae* ortholog.
