# Supplementary material for: Proteomic analysis of protein phosphatase Z1 from Candida albicans
Source: PLoS One. 2017 Aug 24;12(8):e0183176. doi: 10.1371/journal.pone.0183176 (PMC5570430; doi:10.1371/journal.pone.0183176)
Supplement: S4 Table — (PDF) [file pone.0183176.s008.pdf]

S4 Table. Identification of proteins in spots that exhibit significant RuBPS staining intensity changes in the absence of CaPpz1

Protein Name[1]	Protein ID[1]	Systemic gene name[2]	Standard gene name[1]	Protein abbreviation[2]	Spot ID[#]	Fold change	p value
14-3-3 protein homolog	O42766	*C1_03220C*	*BMH1*	Bmh1	47	-1.97	0.006
40S ribosomal protein S0	O42817	*C3_05370C*	*YST1*	Yst1	**22**	-2.35	0.042
60S ribosomal protein L20	A0A1D8PLC9	*C4_01520C*	*RPL20B*	Rpl20B	**10**	-2.21	0.021
Adenylosuccinate synthetase	P0CH96	*C1_09640W*	*ADE12*	Ade12	46	2.60	0.045
Deoxyhypusine hydroxylase	Q59Z14	*C2_07290W*	*LIA1*	Lia1-like**	**18**	-1.59	0.019
Elongation factor 1-beta	A0A1D8PM35	*C4_04480C*	*EFB1*	Efb1	43	-2.37	0.002
Elongation factor 3*	P25997	*C5_01580C*	*CEF3*	Cef3	44	-7.08	0.004
					**27**	-3.63	0.020
Heat shock protein SSA1	P41797	*C1_13480W*	*HSP70*	Hsp70/Ssa1	**32**	-1.52	0.042
Isocitrate dehydrogenase [NAD] subunit 1 (mitochondrial)	A0A1D8PEM5	*C1_09630W*	*IDH1*	Idh1	45	2.76	0.017
Peptidyl-prolyl cis-trans isomerase	Q5ALM6	*C2_02320C*	*CPR3*	Cpr3	**02**	-2.20	0.006
Peroxiredoxin TSA1	Q9Y7F0	*C3_06180C*	*TSA1*	Tsa1	**16**	-2.23	0.002
Protein URE2	Q96WL3	*C2_04710C*	*URE2*	Ure2	48	1.95	0.0004
Transketolase 1	Q5A750	*C1_08320W*	*TKL1*	Tkl1	**29**	1.84	0.012
Ubiquitin-activating enzyme E1	A0A1D8PKJ3	*C3_06500W*	*UBA1*	Uba1	**30**	1.59	0.029

Based on [1]http://www.uniprot.org/ and [2] http://www.candidagenome.org/.
*The protein was identified in two separate spots.
** The abbreviation is based on the name of the *S. cerevisiae* ortholog.
[#] Phosphoprotein containing spots (see S3 Table) are highlighted in bold face.
